# IRE1α/XBP1s branch of UPR links HIF1α activation to mediate ANGII-dependent endothelial dysfunction under particulate matter (PM) 2.5 exposure

**DOI:** 10.1038/s41598-017-13156-y

**Published:** 2017-10-18

**Authors:** Xiuduan Xu, Aodeng qimuge, Hongli Wang, Chen Xing, Ye Gu, Shasha Liu, Huan Xu, Meiru Hu, Lun Song

**Affiliations:** 1 Department of Stress Medicine, Beijing Institute of Basic Medical Sciences, 27 Taiping Road, Beijing, 100850 P. R. China; 2 Anhui Medical University, 81 Meishan Road, Hefei, 230032 P. R. China; 3 Guangxi Medical University, 22 Shuangyong Road, Nanning, 530021 P. R. China; 4 Department of New Drug Screening Center, China Pharmaceutical University, 24 Tongjiaxiang, Nanjing, 210009 P. R. China; 5 Laboratory of Cellular and Molecular Immunology, School of Medicine, Henan University, 357 Ximen Road, Kaifeng, 475004 P. R. China; 6 Department of Pathology, School of Basic Medical Sciences, Lanzhou University, Tianshui South Road, Lanzhou, 730000 P. R. China; 7 Department of Gastroenterology and Hepatology, Chinese PLA, 21 General Hospital, Beijing, China

## Abstract

Short- and long-term exposure to particulate matter (PM) 2.5 instigates adverse health effect upon the cardiovascular (CV) system. Disclosing the molecular events by which PM2.5 evokes CV injuries is essential in developing effective risk-reduction strategy. Here we found that rats after intratracheally instillation with PM2.5 displayed increased circulating level of ANGII, the major bioactive peptide in renin-angiotensin-system (RAS), which resulted from the elevation of ANGII production in the vascular endothelium. Further investigations demonstrated that activation of IRE1α/XBP1s branch of unfolded protein response (UPR) was essential for augmented vascular ANGII signaling in response to PM2.5 exposure, whose effects strictly depends on the assembly of XBP1s/HIF1α transcriptional complex. Moreover, ablation of IRE1/XBP1/HIFα-dependent ACE/ANGII/AT1R axis activation inhibited oxidative stress and proinflammatory response in the vascular endothelial cells induced by PM2.5. Therefore, we conclude that PM2.5 exposure instigates endoplasmic reticulum instability, leading to the induction of IRE1α/XBP1s branch of UPR and links HIF1α transactivation to mediate ANGII-dependent endothelial dysfunction. Identifying novel therapeutic targets to alleviate ER stress and restore local RAS homeostasis in the endothelium may be helpful for the management of PM2.5-induced CV burden.

## Introduction

Air pollution is an important public health issue, causing adverse health effects worldwide. The most harmful component of air pollution, particulate matter (PM), especially the fine particles (aerodynamic diameter <2.5 μm, PM2.5), is strongly associated with severe air pollution-induced health threats. PM2.5 can not only reach the deep parts of the respiratory tract, but also penetrate deeply into the lung alveoli and enter the bloodstream. Consistent evidences from both epidemiological and experimental studies have demonstrated that short- and long-term exposure to PM2.5 is related to respiratory and cardiovascular morbidity and mortality^1–6^. Importantly, acute exposure to PM2.5 resulted in a higher rate of death due to cardiovascular than respiratory diseases (69% cardiovascular versus 28% respiratory)^7^, which suggests that although PM may intuitively exert a damage effect mostly on the lungs, the majority adverse health effect of PM are upon the cardiovascular system.

Numerous studies have consistently demonstrated that acute PM exposure significantly increases clinical manifestations of a host of cardiovascular diseases (CVD), including myocardial infarction and ischemia, strokes, heart failure and arrhythmias; while long-term exposure has been shown to accelerate the progression of atherosclerotic plaques and enhance the chronic development of atherosclerosis^1–6,8^. According to the human, animal and toxicological studies on illustrating the mechanisms by which particle exposure may trigger cardiovascular events, PM2.5 inhaled into the pulmonary tree may instigate remote cardiovascular health effects via inducing release of systemic inflammation and/or oxidative stress mediators from lung-based cells, altering autonomic nervous system imbalance or directly interacting with the vasculature after the soluble particle constituents translocating into CV system. In turn, these responses have been shown to trigger acute arterial vasoconstriction, hypertension, endothelial dysfunction, arrhythmias and pro-coagulant/thrombotic actions^1–6^. Unfortunately, the molecular events involving in PM2.5-associated CVD burden have not been clearly clarified.

The renin-angiotensin system (RAS), one of the most important hormonal systems, plays a major role in the pathophysiology of cardiovascular disorders. Angiotensin II (ANGII), the major bioactive peptide of this system, is known to be involved in the advancement of CVD by inducing vasoconstrictive effects, endothelial dysfunction, inflammatory reactions, thrombosis and oxidative injuries^9–11^. ANGII is obtained from its precursor molecule, angiotensinogen (AGT), which is primarily synthesized in the liver and adipose tissue, and to a lesser extent in the kidney, brain, heart, adrenal, and vascular walls. AGT is first converted by renin to produce a decapeptide, angiotensin I (ANGI), which is then converted to ANGII by removal of a COOH-terminal dipeptide by angiotensin-converting enzyme (ACE). ANGII primarily mediates its action through the ANGII type 1 receptor (AT1R) subtype, a 7-transmembrane G-protein-coupled receptor expressed widely and coupled to various intracellular signaling molecules after activation. It is well-accepted that inappropriate elevation of circulating/systemic or local/tissue ACE/ANGII/AT1R axis components results in RAS-related injuries, including initiation and progression of CVD^9–11^. However, the exact source and the according mechanism responsible for circulating and local augmented RAS components under various pathophysiological conditions remains underdetermined.

Endoplasmic reticulum (ER) stress, together with the unfolded protein response (UPR), plays a critical role in the maintenance of ER homeostasis and the pathogenesis of a variety of human diseases. The UPR is distinguished by the action of three transmembrane proteins named inositol-requiring enzyme 1α (IRE1α), protein kinase RNA-like ER kinase (PERK) and activating transcription factor 6 (ATF6). These sensor proteins are able to induce three arms of UPR under ER stress, including PERK activation-dependent eukaryotic translational initiation factor 2α (eIF2α) phosphorylation, IRE1 activation-dependent X-box-binding protein 1 (XBP1) splicing and the cleavage of ATF6 associated with Golgi apparatus. These three branches of UPR have been found to be implicated in the regulation of cardiovascular disorders, such as atherosclerosis, cardiac hypertrophy, ischemia heart disease and heart failure, either by destructive or protective actions^12–16^.

In our previous study, we have disclosed that PM2.5 collected in Wuhan, a southern city in China, evokes airway inflammation by inducing autophagy-dependent proinflammatory cytokine production in the bronchial epithelial cells^17^. In the current study, we further demonstrated that Wuhan PM2.5 exposure induced elevation of systemic ANGII and local ACE/ANGII/AT1R axis activation and the subsequent oxidative stress and proinflammatory responses in the vascular endothelium. Most importantly, IRE1α/XBP1s branches of UPR links hypoxia-inducible factor 1α (HIF1α) activation to mediate the local augmented RAS components and the endothelial dysfunction ensued. These novel findings not only provide previously unidentified evidence linking ER stress with particle toxicology, but also elucidate new mechanism for controlling vascular RAS activities by UPR-dependent transcriptional events and its contribution to PM-induced CV pathologies.

## Results

### PM2.5 exposure resulted in elevated plasma concentration of ANGII and augmented ACE/ANGII/AT1R axis components in rat vascular endothelial cells

**Figure 1:**
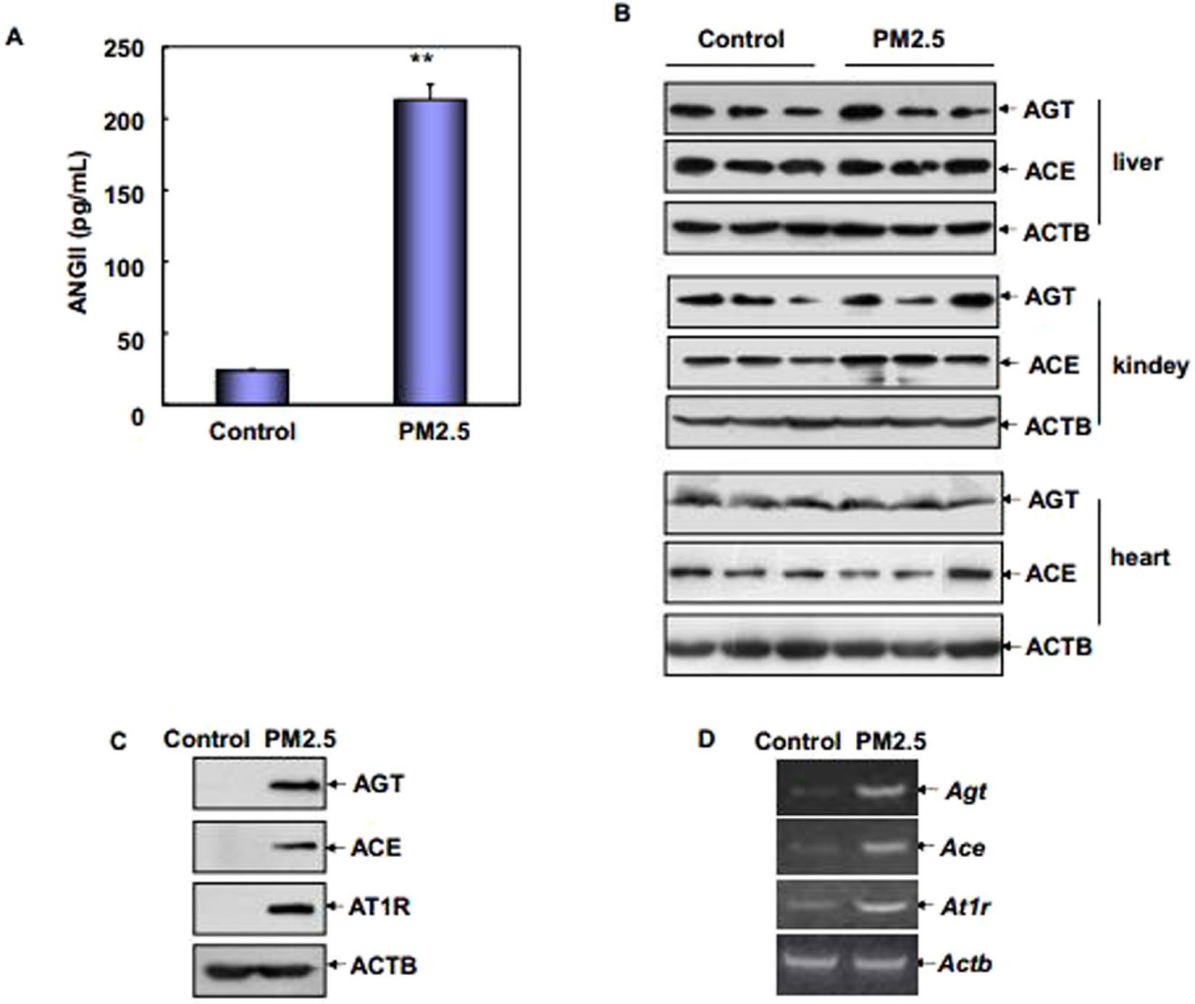
PM2.5 exposure induced augmented serum ANGII and ACE/ANGII/AT1R axis components in rat vascular endothelial cells. (**A**) The serum level of ANGII was detected in SD rats IT instilled with PM2.5 or identical volume of sterile saline by ELISA (**P < 0.01). (**B**) Both AGT and ACE levels were detected in liver, kindey and heart of control and PM2.5-treated rats. The experimental results of 3 representative rats in each group were presented (**C**,**D**) The aortic endothelial cells were isolated from the above PM2.5-treated and control rats and then the transcription and protein synthesis of ACE/ANGII/AT1R axis components were determined, respectively.

In order to test the hypothesis that RAS, the well-recognized key regulators of CV disorders, could be playing an important role in mediating the health risk effect of airborne particles on CV system, we first analyze the changes on the systemic and tissue-specific RAS components in the SD rats exposed to Wuhan PM2.5. As shown in Fig. 1A, inhalation of PM2.5 led to significant elevation of plasma concentration of ANGII, the major bioactive peptide of RAS. To further determine the potential source responsible for circulating augmented ANGII, we next detected the levels of AGT and ACE, the major components of RAS which are necessary for generation of ANG II, in the liver, kidney, heart, lung and vascular endothelium. We found that local levels of AGT and ACE did not show detectable changes in the liver, kidney, heart (Fig. 1B) and lung (no signal) of the control and PM2.5-treated rats, but the levels of these RAS components dramatically increased in the vascular endothelium after pulmonary PM2.5 exposure (Fig. 1C). Moreover, local expression level of AT1R also upregulated in response to PM2.5 inhalation in the vascular endothelium, but not in other tissues (Fig. 1B and C). RT-PCR assay further showed that the *Agt, Ace* and *At1r* mRNA were constitutively expressed in the vascular endothelium and their levels displayed a significant increase upon PM2.5 exposure conditions (Fig. 1D). These data indicate that the transcription of ACE/ANGII/AT1R axis components is highly enhanced in the rat vascular endothelium that was stressed by PM2.5 exposure, which cause a parallel increase in the formation of serum ANGII.

### *In vitro* PM2.5 exposure promoted ACE/ANGII/AT1R axis components expression in HUVECs and RAEC

**Figure 2:**
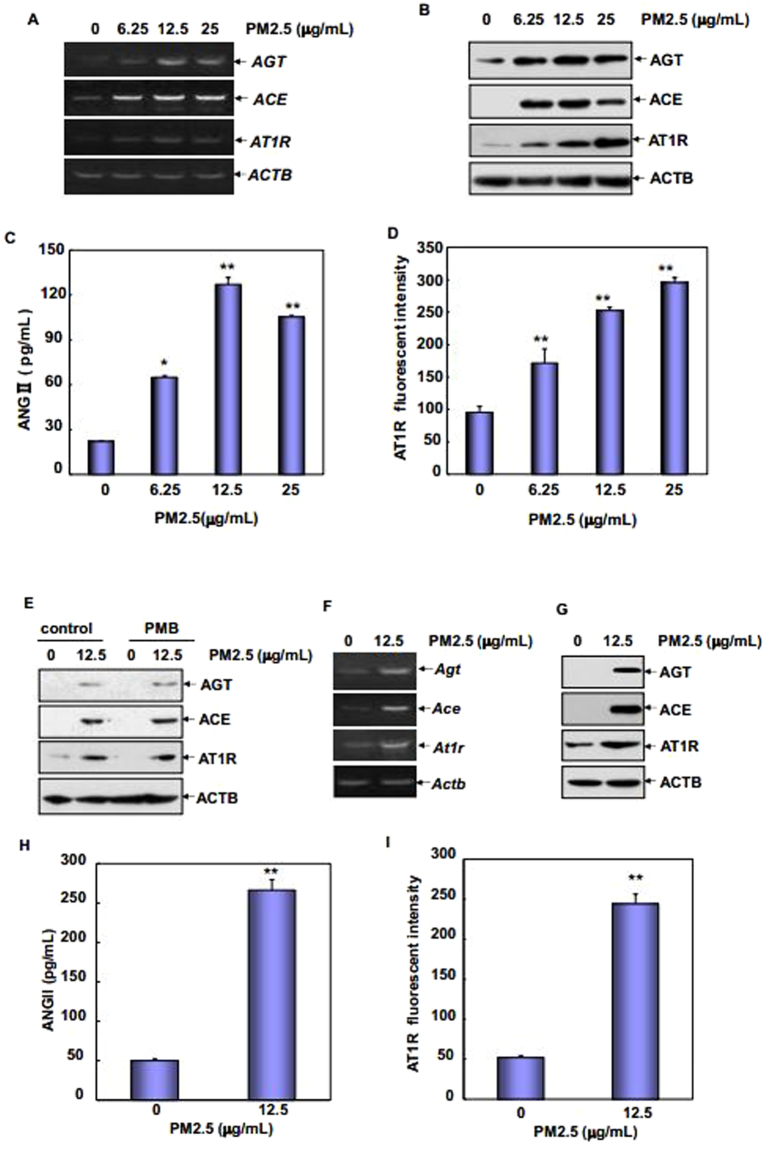
*In vitro* PM2.5 exposure upregulated ACE/ANGII/AT1R axis components expressions in HUVECs and RAECs. (**A**,**B**) HUVECs were treated with different doses of PM2.5 for 24 hr and then the transcription and protein synthesis of AGT, ACE and AT1R were determined. (**C,D**) HUVECs were treated with PM2.5 for 24 hr and then ANGII production in the cell supernatants and cell surface level of AT1R were determined by ELISA and flow cytometric assay, respectively (**P < 0.01). (**E**) HUVECs were left untreated or treated with PM2.5 alone or in combination with PMB (50 μg/mL) for 24 hr. Then, the induction of AGT, ACE and AT1R expression was determined. (**F**–**I**) The aortic endothelial cells were isolated from SD rats and then subjected to PM2.5 exposure for 24 hr. The transcription and protein synthesis of AGT, ACE and AT1R, ANGII production in the cell supernatants and cell surface level of AT1R were determined (**P < 0.01).

To further confirm the effect of PM2.5 on the regulation of RAS components generation in the vascular endothelium, *in vitro* assays were performed by using the human umbilical vein endothelial cells (HUVECs) and the freshly isolated primary rat aortic endothelial cells (RAECs). Firstly, HUVECs were treated with different doses of PM2.5 and then the expressions of AGT, ACE and AT1R were determined after PM2.5 exposure. As shown in Fig. 2A and B, *AGT, ACE* and *AT1R* transcription as well as their protein synthesis were significantly upregulated following PM2.5 treatment at different doses. Under the same conditions, generation of ANGII in the supernatants of cultured HUVECs and cell surface level of AT1R were also dramatically increased, and the peak induction of these components were observed upon 12.5~25 μg/mL of PM2.5 exposure (Fig. 2C and D). To exclude the possibility that PM2.5 exposure leads to a general activation of gene transcription and protein synthesis in HUVECs, here we provided more evidence to show that the expression levels of several other signaling molecules, such as ERK, p38K and ATF2, did not show detectable changes before and after PM2.5 exposure (Supplementary Fig. S1A). In addition, no obvious difference was observed when the amount of total proteins in the untreated and PM2.5-treated HUVECs was detected (Supplementary Fig. S1B). These data suggest that upregulation of RAS components expression in HUVECs were a specific response to PM2.5 exposure, but did not resulted from the increase of bulk rate of protein syntheses. Moreover, induction of these three RAS components in response to PM2.5 exposure did not show any changes in the absence or presence of the co-treatment of PMB, an antibiotic widely used to eliminate the effects of endotoxin contamination (Fig. 2E). These data together indicate that PM2.5 exposure exerts a specific effect to upregulate ACE/ANGII/AT1R axis components expression in HUVECs, which response is unrelated to endotoxin contamination.

Next, RAECs were isolated and then subjected to PM2.5 exposure. Similarly, an induction of *Agt, Ace* and *At1r* transcription and an increase in these components protein synthesis were observed after PM2.5 treatment (Fig. 2F and G). Furthermore, elevated production of ANGII in the supernatants of cultured RAECs (Fig. 2H) and enhancement of cell surface level of AT1R was detected in response to PM2.5 treatment (Fig. 2I). These data therefore further confirm that PM2.5 exposure is capable of inducing upregulation of ACE/ANGII/AT1R axis components in the vascular endothelial cells.

### IRE1α/XBP1s branch of UPR was responsible for augmented ACE/ANGII/AT1R axis components in vascular endothelial cells under PM2.5 exposure

**Figure 3:**
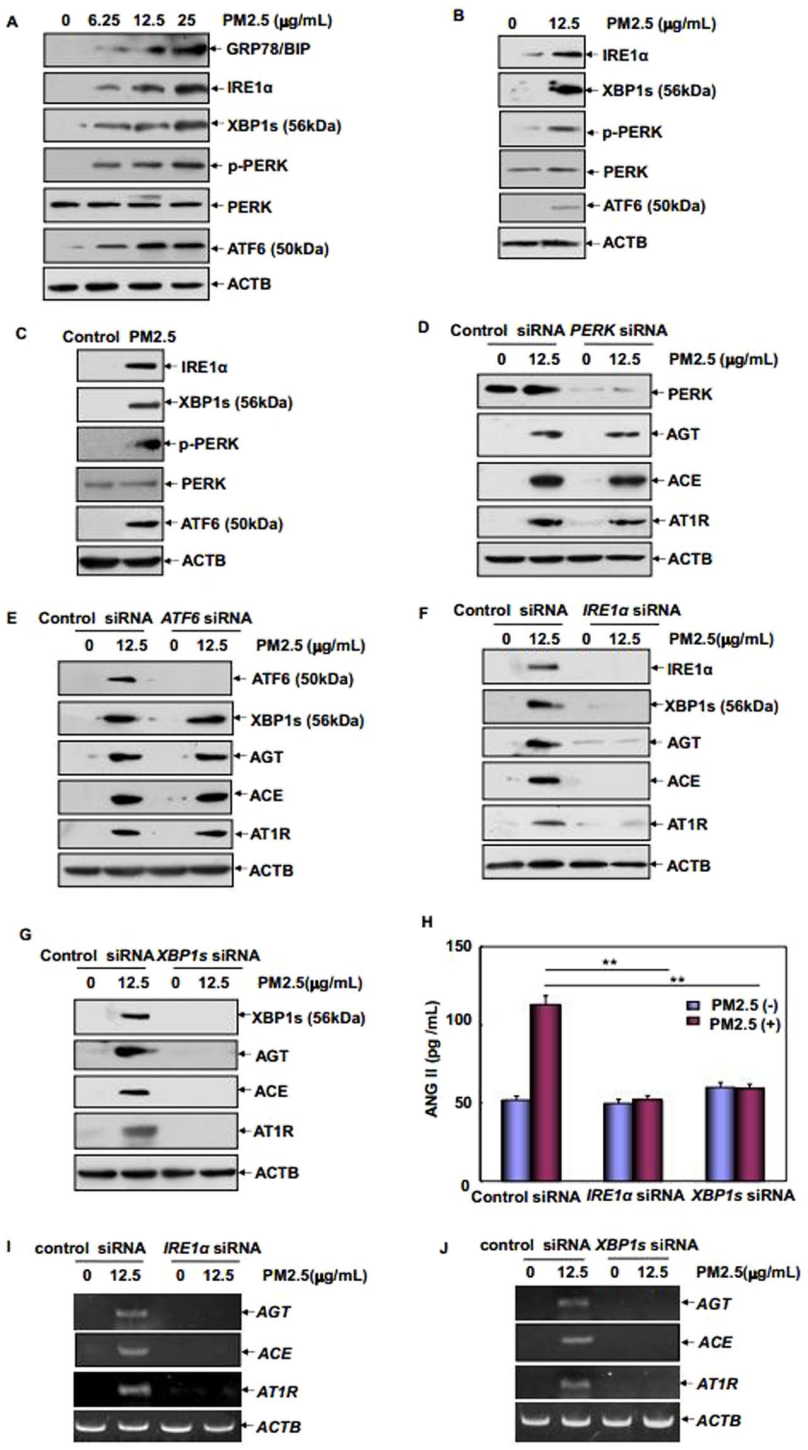
IRE1α/XBP1s branch of UPR was responsible for augmented ACE/ANGII/AT1R axis components in vascular endothelial cells under PM2.5 exposure. (**A**) HUVECs were treated with different doses of PM2.5 and then the activation of three branches of UPR was determined. (**B**) The isolated RAECs were cultured and treated as described in 2 F and then the activation of three branches of UPR was determined. (**C**) The activation of three branches of UPR was determined in the aortic endothelial cells isolated from PM2.5-treated or control rats. (**D**–**G**) HUVECs were transfected with the siRNAs targeting three branches of UPR and then treated with PM2.5. The expression of AGT, ACE, AT1R, and XBP1s were determined 24 hr after PM2.5 exposure. (**H**–**J**) HUVECs were transfected with the indicated siRNAs and then subjected to PM2.5 exposure for 24 hr. Then ANGII production in cell supernatants and the transcription of *AGT, ACE* and *AT1R* mRNA were determined (**P < 0.01).

We next focused on elucidating the intracellular signaling events leading to upregulation of ACE/ANGII/AT1R axis components expression in the vascular endothelium under PM2.5 exposure. Since ER stress and UPR play important roles in the initiation and progression of CV pathologies^12–16^, we thus detected whether UPR is involved in RAS components upregulation in the PM2.5-treated vascular endothelial cells. As shown in Fig. 3A, PM2.5 exposure induced a dose-dependent increase of GRP78/BIP, IRE1α, and ATF6 expression as well as PERK phosphorylation in HUVECs. Under the same conditions, we did not observed the expression of the unspliced form of XBP1 (XBP1u) protein in HUVECs before and after PM2.5 treatment, but a significant upregulation of the spliced form of XBP1 (XBP1s) expression was readily detected (Fig. 3A). Consistently, an increase followed by a decrease in *XBP1u* mRNA and a consistent increase in *XBP1s* mRNA expression were observed in HUVECs after PM2.5 exposure (Supplementary Fig. S2). These data indicate that three branches of UPR could be activated in HUVECs under PM2.5 exposure. In the following study, we also observed the activation of all three arms of UPR in both cultured RAECs in response to PM2.5 exposure (Fig. 3B) and the fresh endothelial cells isolated from PM2.5-treated SD rats (Fig. 3C). These *in vitro* and *in vivo* assays further confirmed the involvement of UPR in the effects of PM2.5 on the vascular endothelial cells. Then, siRNAs targeting three branches of UPR were separately transfected into HUVECs. As shown in Fig. 3D and E, no obvious changes on PM2.5-induced AGT, ACE and AT1R expressions were observed in the absence or presence of PERK and ATF6 activation; while the augmented expression of these RAS components under PM2.5 exposure was almost totally blocked when IRE1α or XBP1s induction was interrupted (Fig. 3F and G). Furthermore, the increase in ANGII generation induced by PM2.5 was also impaired in both IRE1α and XBP1s siRNA-transfected HUVECs (Fig. 3H). Most importantly, knockdown the expression of IRE1α, but not ATF6, blocked the induction of XBP1s expression (Fig. 3E and F). These data together indicate that IRE1α functions as the upstream activator for XBP1 splicing and the IRE1α/XBP1s branch of UPR is responsible for augmented ACE/ANGII/AT1R axis components in vascular endothelial cells under PM2.5 exposure.

Since AGT, ACE and AT1R abundance were regulated at the transcriptional level in the vascular endothelium under PM2.5 exposure (Figs 1 and 2); we next compared the mRNA transcription of these RAS components in the absence or presence of IRE1α/XBP1s pathway activation. As shown in Fig. 3I and J, blocking IRE1α/XBP1s arm of UPR dramatically inhibited the induction of *AGT, ACE* and *AT1R* transcription in response to PM2.5 exposure, which was consistent with the suppression of these proteins induction under the same experimental conditions (Fig. 3F and G). These data together demonstrate that IRE1α/XBP1s pathway activation is implicated in the elevation of transcription and protein synthesis of ACE/ANGII/AT1R axis components and local RAS activation in the vascular endothelium in response to PM2.5 exposure.

### IRE1α/XBP1s pathway linked HIF1α to mediate augmented ACE/ANGII/AT1R axis components in vascular endothelial cells under PM2.5 exposure

**Figure 4:**
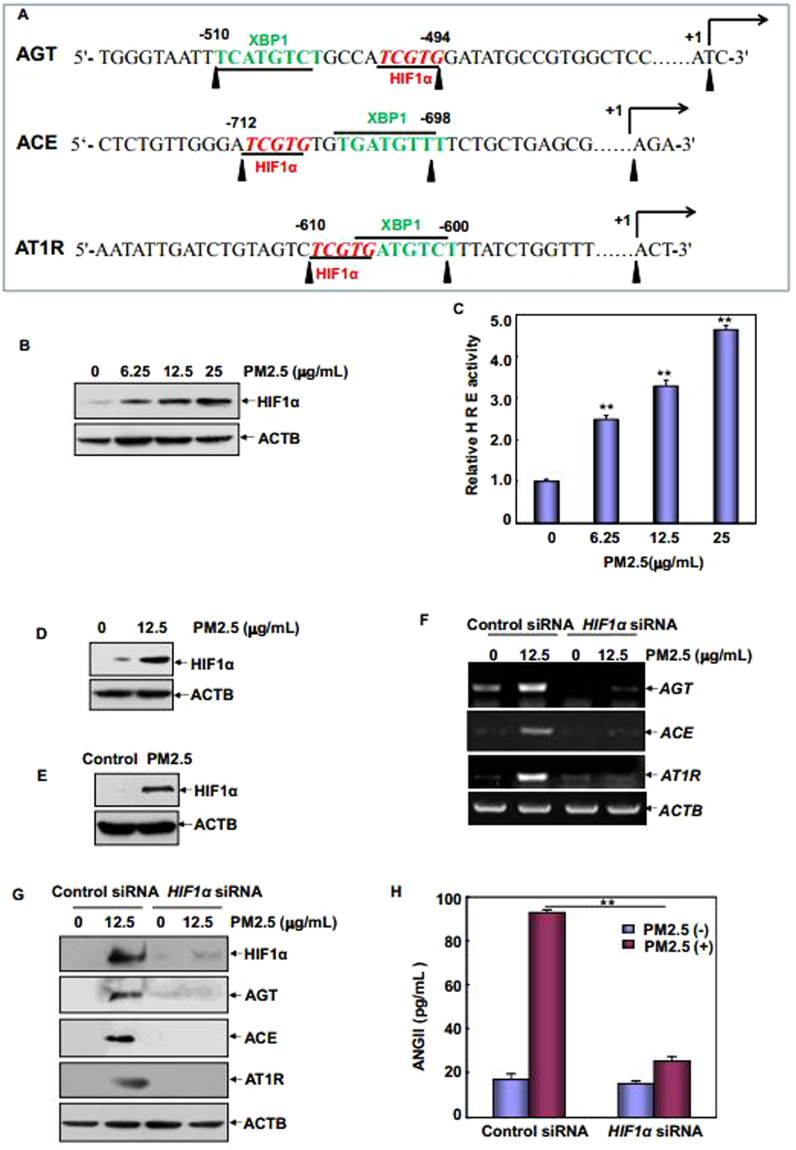
HIF1α was accumulated and responsible for ACE/ANGII/AT1R axis components induction in the vascular endothelium under PM2.5 exposure. (**A**) An analysis of human *AGT, ACE* and *AT1R* promoters indicated the potential XBP1s and HIF1α-responsive elements located closely within the promoter regions of these three genes. (**B**) HUVECs were treated with different doses of PM2.5 for 24 hr and then the accumulation of HIF1α was determined. (**C**) HUVECs were transfected with a HRE-driven luciferase reporter and then exposed to different doses of PM2.5. The induction of HRE-dependent luciferase activity was determined 24 hr after PM2.5 exposure (**P < 0.01). (**D**) The isolated RAECs were cultured and treated as described in Fig. 2F and the accumulation of HIF1α was determined. (**E**) The accumulation of HIF1α was determined in the aortic endothelial cells isolated from PM2.5-treated or control rats. (**F**–**H**) HUVECs were transfected with the indicated siRNAs and then treated with PM2.5 for 24 hr. The transcription and protein synthesis of AGT, ACE and AT1R and ANGII production were determined (**P < 0.01).

To further address the detailed mechanism involving in the transcriptional control of ACE/ANGII/AT1R axis components by IRE1α/XBP1s arm of UPR in the PM2.5-treated vascular endothelial cells, an analysis of -1000 bp region of human and rat *AGT, ACE* and *AT1R* promoter was performed to find if there are any clusters of cis-acting DNA regulatory elements, perfectly or partially matched to binding sequence for XBP1s (T*C/G*ATGT*C/T*T) (http://jaspar.genereg.net/). Fortunately, potential XBP1s-responsive elements were respectively identified in both human (Fig. 4A) and rat (data not shown) *AGT, ACE* and *AT1R* promoters. Interestingly, we also found binding sites for HIF1α close to or overlapping with XBP1s-responsive elements within the promoter regions of these three genes (Fig. 4A). So we speculated that XBP1s may drive the transcription of ACE/ANGII/AT1R axis components by assembling a transcriptional complex with HIF1α. To address this possibility, we firstly determined if HIF1α contributed to the transcriptional induction of ACE/ANGII/AT1R axis components. As shown in Fig. 4B and C, PM2.5 treatment induced a dose-dependent accumulation of HIF1α and elevation of hypoxia responsive elements (HRE)-driven luciferase activities in HUVECs, indicating that HIF1α could be activated in response to PM2.5 exposure. Induction of HIF1α expression in both cultured RAECs after PM2.5 exposure and the fresh vascular endothelial cells isolated from PM2.5-treated SD rats further confirmed the involvement of HIF1α in the effects of PM2.5 on the vascular endothelial cells (Fig. 4D and E). Moreover, when HIF1α induction was suppressed, both the transcription and the protein synthesis of AGT, ACE and AT1R were totally blocked in PM2.5-treated HUVECs (Fig. 4F and G). Under the same experimental conditions, ANGII production induced by PM2.5 was also significantly inhibited (Fig. 4H). Taken together, we conclude that HIF1α is involved in the transcriptional induction of ACE/ANGII/AT1R axis components in the vascular endothelial cells under PM2.5 exposure.

**Figure 5:**
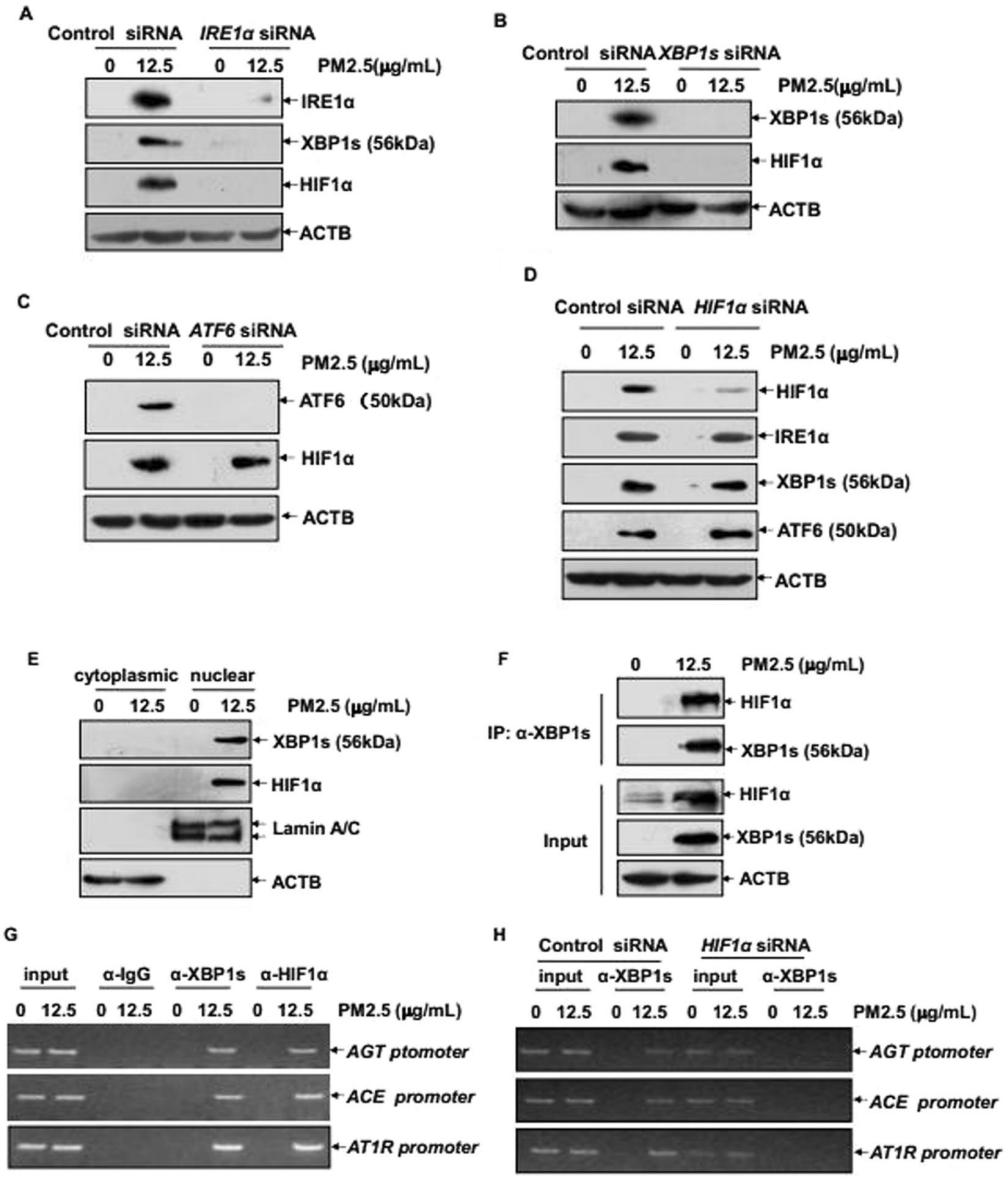
IRE1α/XBP1s pathway activation linked HIF1α to mediate ACE/ANGII/AT1R axis components upregulation in the vascular endothelium under PM2.5 exposure. (**A**–**D**) HUVECs were transfected with the indicated siRNAs and then subjected to PM2.5 exposure. The induction of IRE1α, XBP1s or HIF1α expressions was determined 24 hr after PM2.5 exposure. (**E**) HUVECs were left untreated or treated with PM2.5 and then the cytoplasmic and nuclear proteins were extracted 24 hr after PM2.5 exposure. The sub-cellular distribution of XBP1s or HIF1α was detected. (**F**) Cell lysate from untreated and PM2.5-treated HUVECs were immunoprecipitated with an anti-XBP1s antibody, and the immunoprecipitants were analyzed with an anti-HIF1α antibody. (**G**) Soluble chromatin was prepared from HUVECs either untreated or treated with PM2.5 and then ChIP assay was performed to detect the binding ability of XBP1s and HIF1α within the human *AGT, ACE* and *AT1R* promoters. (**H**) HUVECs were transfected with *HIF1α* or control siRNA and then exposed to PM2.5. ChIP assay was performed to compare the binding ability of XBP1s within the indicated promoters with or without the aid of HIF1α.

Next, we tried to figure out the functional link between XBP1s and HIF1α in PM2.5-induced response in the vascular endothelial cells. As shown in Fig. 5A and B, knockdown either IRE1α or XBP1s expression completely blocked HIF1α accumulation in HUVECs under PM2.5 exposure. However, no detectable changes on HIF1α accumulation were observed with the impairment of ATF6 expression (Fig. 5C). Furthermore, suppressing HIF1α accumulation did not affect the induced expression or activation of any ER sensor proteins in HUVECs (Fig. 5D). These data indicate that HIF1α functions as the downstream target of IRE1α/XBP1s pathway in mediating RAS components expression in the PM2.5-treated vascular endothelial cells. In the following study, we observed the accumulation of both XBP1s and HIF1α within the nucleus (Fig. 5E), and an induction of XBP1s/HIF1α complex was formed in HUVECs after PM2.5 exposure (Fig. 5F). Moreover, a strong association of XBP1s and HIF1α to the same chromatin regions of *AGT, ACE* and *AT1R* promoter was readily detected according the results from ChIP assay (Fig. 5G). Most importantly, without the aid of HIF1α, XBP1s lost its ability to recruit to the promoter region of these three RAS component genes (Fig. 5H). Taken together, we conclude that activation of XBP1s by IRE1α not only is required for the induction of HIF1α accumulation, but can also recruit to the promoter regions of ACE/ANGII/AT1R axis components by binding with HIF1α and then cooperatively trigger the synthesis of these RAS components under PM2.5 exposure.

### ANGII/AT1R cascade activation mediated PM2.5-induced proinflammatory and oxidative stress responses in the vascular endothelial cells

**Figure 6:**
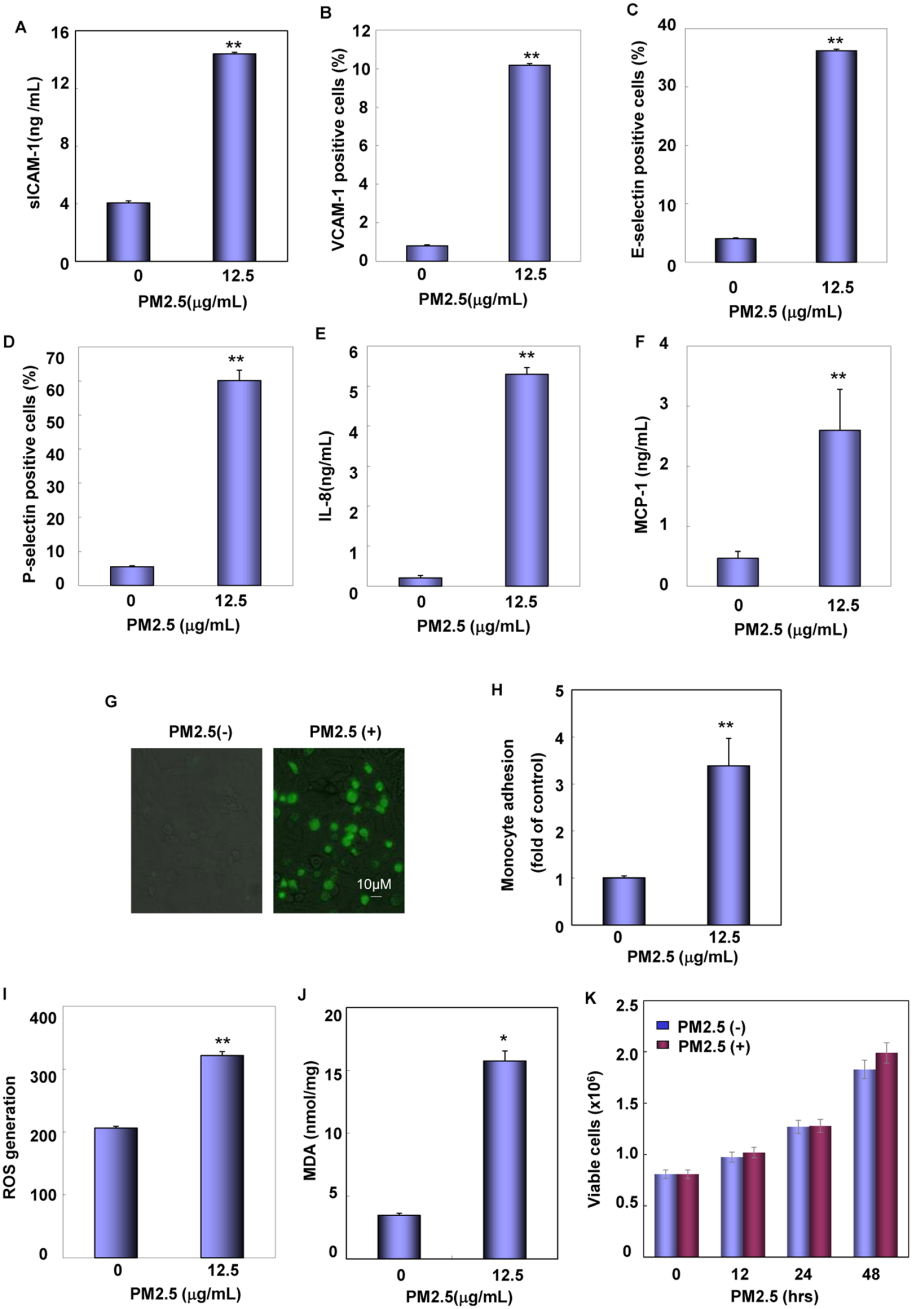
PM2.5 exposure induced proinflammatory and oxidative stress responses in the vascular endothelial cells. (**A**–**F**) HUVECs were treated with PM2.5 (12.5 μg/mL) for 24 hr and then surface levels of adhesive molecules and production of chemokines were determined (**P < 0.01). (**G**–**H**) PM2.5-treated or untreated HUVECs were co-cultured with BCECF/AM-labeled U937 cells. The adhesion of U937 cells to the endothelial cells were evaluated by photography under the confocal microscopy or quantitative flow cytometry (**P < 0.01). (**I,J**) HUVECs were treated with PM2.5 for 24 hr and then generation of ROS and MDA were determined (**P < 0.01). (**K**) HUVECs were treated with PM2.5 for the indicated time periods and then the number of viable cells was tested by trypan blue exclusion essay.

After disclosing the upstream molecular basis for the elevation of ACE/ANGII/AT1R axis components expression under PM2.5 exposure, we next focused on examining whether augmented systemic and local ANGII could exert any downstream pathological effect through its receptor, AT1R, in the vascular endothelial cells. Since inappropriate ANGII/AT1R pathway activation plays a critical role in the initiation and maintenance of endothelial dysfunction mainly by triggering proinflammatory and oxidative stress responses^11^, we therefore detected the expressions of chemokines and cell adhesion molecules driving vascular inflammation and the regulators of oxidative stress responses in HUVECs under PM2.5 exposure. According to the results from ELISA and flow cytometric assays, we found higher level of VCAM-1, E-selectin and P-selectin on the surface and more sICAM-1, IL-8 and MCP-1 in the supernatants of HUVECs after PM2.5 treatment (Fig. 6A–F). Under the same conditions, a significant increase in adhesion of U937 monocytes to HUVECs after PM2.5 exposure was readily observed in the co-culture assay (Fig. 6G and H). These data indicate that PM2.5 is potent in upregulating the expression of chemokines and cell adhesion molecules in the vascular endothelial cells, which subsequently recruits monocytes and thus induces a proinflammatory response in the vascular endothelium. In the following study, we also found the elevation of ROS and MDA production in PM2.5-treated HUVECs; while the cell viability did not show detectable difference at the presence or absence of PM2.5 exposure (Fig. 6I–K). These data suggest that PM2.5 exposure is also capable to induce an oxidants/antioxidants imbalance in the vascular endothelial cells without influence to the survival of cells.

**Figure 7:**
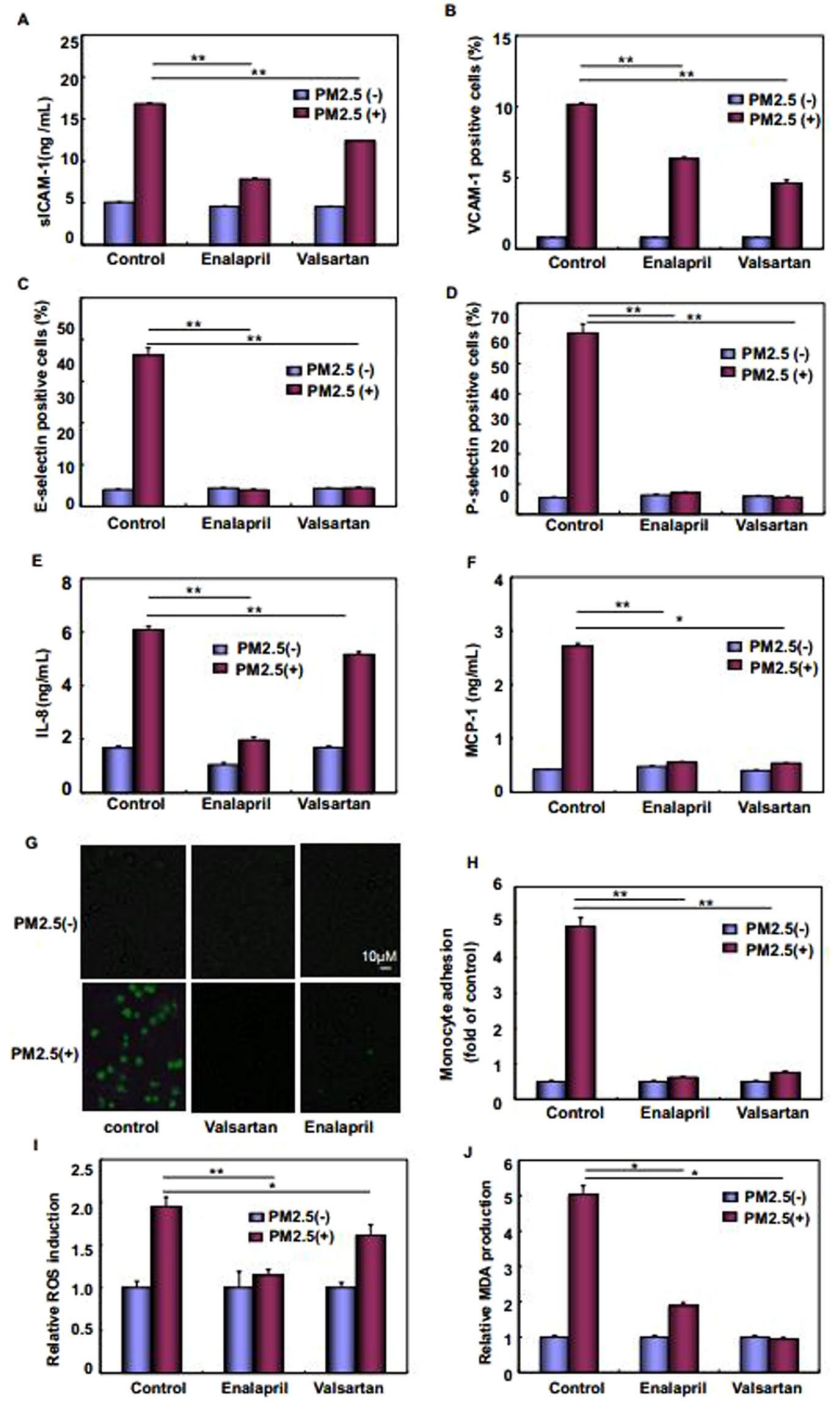
Suppressing ANGII/AT1R cascade activation alleviated proinflammatory response and oxidants/antioxidants imbalance induced by PM2.5. HUVECs were pretreated with enalapril (100 μM) or valsartan (1 μM) for 2 hr and then subjected to PM2.5 exposure for 24 hr. The surface levels of adhesive molecules and production of chemokines (**A**–**F**), adhesion of U937 cells to HUVECs (**G**,**H**) and generation of regulators for oxidative stress responses (**I,J**) were determined (**P < 0.01, *P < 0.05).

In order to address whether ANGII/AT1R pathway activation plays any role in mediating the pro-inflammatory and oxidative stress responses in the vascular endothelial cells induced by PM2.5, HUVECs were pretreated with either enalapril, the ACE inhibitor, or valsartan, the AT1R antagonist, followed by exposure to PM2.5. After confirming the efficiency of these chemicals in inhibiting ANGII production and ANGII/AT1R pathway activation in response to PM2.5 exposure (Supplementary Fig. S3), the changes on the signaling molecules involving in vascular injuries were determined in HUVECs. We found that interrupting ANGII/AT1R cascade activation resulted in downregulation of VCAM-1, E-selectin, P-selectin, sICAM-1, IL-8, and MCP-1 synthesis to different extent (Fig. 7A–F), and the induced adhesion of U937 monocytes to HUVECs was nearly total blocked under the same PM2.5 exposure conditions (Fig. 7G and H). Meanwhile, we also observed the suppression of ROS and MDA generation with the impairment of ANGII/AT1R axis activation in PM2.5-treated HUVECs (Fig. 7I and J). These data together indicate that augmented systemic and local ANGII strongly induces proinflammatory and oxidative stress responses by activating AT1R, and thus exacerbating endothelial dysfunction under PM2.5 exposure. It is worth noting that inhibiting ANGII production or blocking AT1R activation effectively suppressed IRE1/XBP1s/HIF1α cascade activation in HUVECs induced by PM2.5 exposure, but the activation states of PERK and ATF6 did not show detectable difference under the same conditions (Supplementary Fig. S3). These results indicate that a positive feed-back regulation between RAS and IRE1/XBP1s/HIF1α axis exists in PM2.5-treated HUVECs.

### The IRE1α/XBP1s/HIF1α signaling pathway activation was critical for mediating ANGII-dependent endothelial dysfunction under PM2.5 exposure

**Figure 8:**
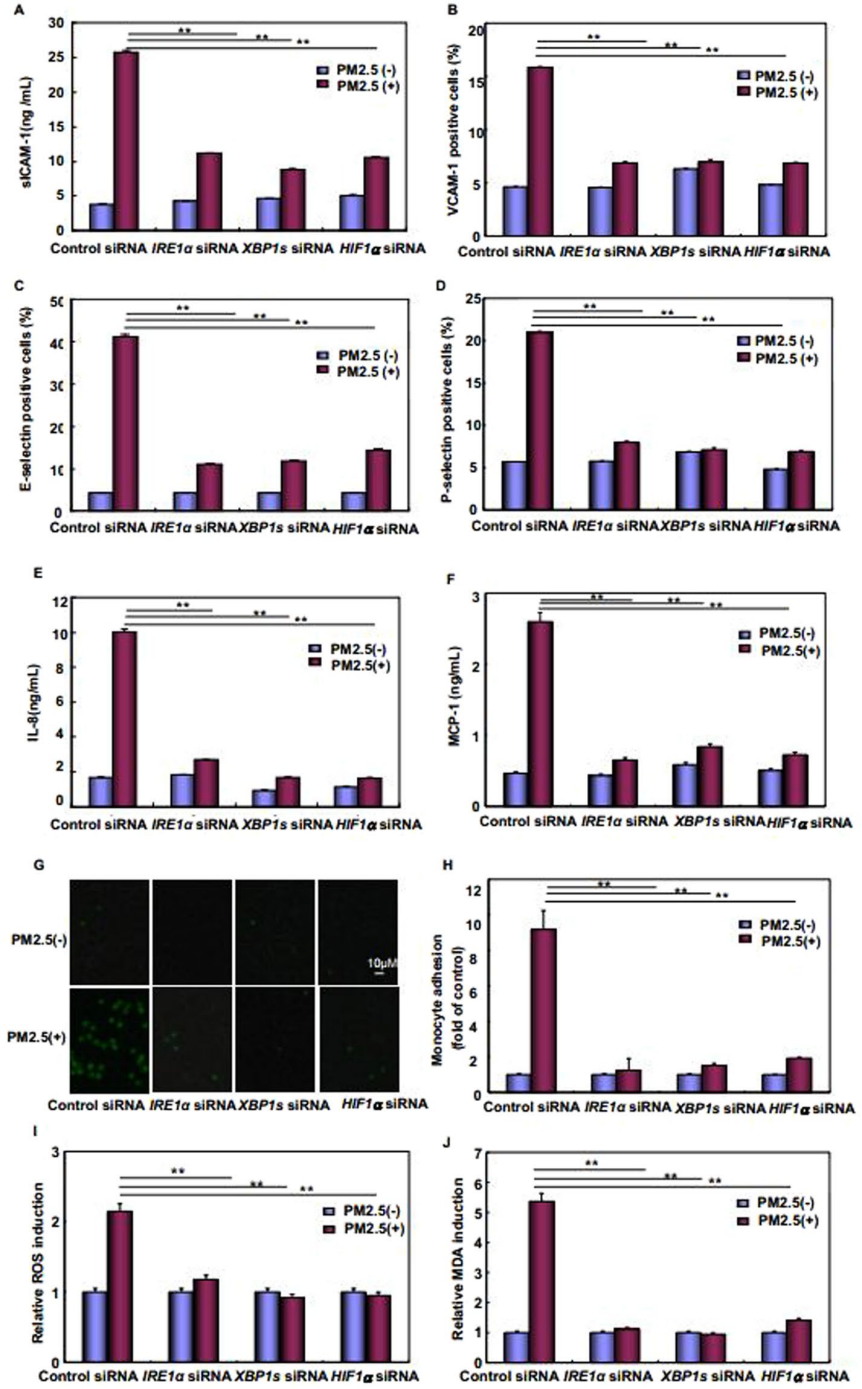
Blocking IRE1α/XBP1s/HIF1α pathway activation attenuated ANGII-dependent endothelial dysfunction under PM2.5 exposure. HUVECs were transfected with the indicated siRNAs and then subjected to PM2.5 exposure for 24 hr. The surface level of cell adhesive molecules and production of chemokines (**A–F**), adhesion of U937 cells to HUVECs (**G,H**) and generation of regulators for oxidative stress responses (**I,J**) were determined (**P < 0.01, *P < 0.05).

Finally, we tried to determine whether IRE1α/XBP1s/HIF1α pathway activation, which mediates augmented ACE/ANGII/AT1R axis components, also contributes to regulating ANGII-dependent endothelial dysfunction under PM2.5 exposure. To this end, IRE1α, XBP1s or HIF1α siRNAs were separately transfected into HUVECs to block the activation of this cascade at different levels. We observed that knockdown of any critical signaling molecules in the IRE1α/XBP1s/HIF1α pathway significantly inhibited proinflammatory mediators expression (Fig. 8A–F) as well as the adhesion of U937 cells to HUVECs induced by PM2.5 (Fig. 8G and H). Under the same conditions, production of ROS and MDA was also dramatically suppressed (Fig. 8I and J). Taken together, these data indicate that the IRE1α/XBP1s/HIF1α pathway activation is critical for mediating ANGII-dependent endothelial dysfunction under PM2.5 exposure.

## Discussion

The epidemiologic association between pulmonary exposure to ambient particulate matter and cardiovascular dysfunction is well known, but the systemic mechanisms that drive this effect remain far from being clarified. In the current study, we have identified the augmentation of systemic ANGII and vascular ACE/ANGII/AT1R axis in mediating endothelial dysfunction in response to acute exposure to PM2.5. Since abnormal activity of systemic and local ANGII signaling have been shown to play a wide role in the pathophysiology of cardiovascular disorders, such as vasoconstriction, endothelial dysfunction, inflammatory reactions, thrombosis, oxidative injuries, etc^9–11^, our results thus may provide novel ANGII-related mechanistic evidence for the epidemiologic studies on short and long time PM2.5 exposure-induced CV pathologies, including hypertension, myocardial infarction, stroke, congestive heart failure and atherosclerosis.

Although it is well-accepted that ANGII precursor, AGT, is primarily synthesized in the liver, kidney, heart and other tissues under physiological conditions^9–11^, we have identified that elevated serum ANGII comes from increased ACE and ANGII production in the vascular endothelium after PM2.5 exposure. Most importantly, local levels of RAS components did not show detectable changes in the liver, kidney, heart or lung in the control and PM2.5-treated rats. These interesting findings suggest that inhaled PM2.5 or the soluble particle constituents may translocate into CV system and directly interact with the vasculature to activate ANGII signaling in the vascular endothelium without causing the same antecedent response in the lung or interfering with the local RAS activity in other tissues. In fact, contribution of endothelium-releasing mechanism to particle-induced CV pathologies has been reported in the previous human and animal studies, which was mediated by the endothelium-derived vasoactive factors, such as endothelin-1 (ET-1) and matrix metallopeptidase 9 (MMP9), and endothelium-dependent inflammatory events^19–22^. And the results form Li, *et al*. has even characterized the clue of RAS involvement in the particle-induced vasoconstriction according to the data from *in vitro* studies by using the AT1R antagonist^23^. However, no previous study provides any *in vivo* and direct evidence for the alteration of systemic or local ANGII in response to urban particles. Therefore, data in the current study firstly confirm the contribution of vascular endothelium-derived ANGII in triggering PM-induced CV pathologies. In fact, although we showed the data of increase on systemic and local ANGII after 7 days of PM2.5 exposure in this study (Fig. 1), particles could induce a rapid response on vascular and circulating ANGII levels immediately within 24 hr (data not shown). Therefore, whether PM2.5 exposure causes a progressive and sustained response in ACE/ANGII/AT1R axis activation is under current investigations.

According to the data from enalapril and valsartan-pretreated cells (Fig. 7), we speculate that keeping homeostasis in the vascular endothelium by targeting ANGII signaling may be helpful for the management of CV burden induced by PM2.5 exposure. But it is worth noting that only pretreatment of the endothelial cells with the inhibitors of ANGII/AT1R pathway is critical for antagonizing vascular dysfunction induced by PM2.5; while enalapril and valsartan treatments followed by PM2.5 exposure could not alleviate the ACE/ANGII/AT1R axis activation and the subsequent proinflammatory and oxidative stress responses in the vascular endothelial cells (data not shown). These data indicate that intervention procedures targeting ANGII signaling only can be effective to avoid vascular dysfunction by performing prior to PM2.5 exposure.

To control the disorder of systemic and vascular ANGII signaling activation, we believe it is imperative to figure out the responsible upstream signal transduction pathway(s) to mediate the augmentation of vascular ACE/ANGII/AT1R axis induced by PM2.5. It is generally approved that AGT abundance is predominantly regulated at the transcriptional level. Several cis-acting DNA regulatory elements have been found in the promoter region of *AGT* gene and the transcription factors which are important in the regulation of *AGT* transcription under both steady state and stress conditions have been identified^24–29^; while few data are reported on the transcriptional control of *ACE* and *AT1R*. Since ER stress and UPR play important roles in the initiation and progression of CV pathologies^12–16^ and there is no previous report on the involvement of UPR in the toxic effects of PM2.5, we thus focused our initial investigation on whether UPR participates in RAS components expression in the PM2.5-treated vascular endothelial cells. To our knowledge, we have for the first time identified the XBP1s-binding site within the promoter regions of *AGT, ACE* and *AT1R* genes and subsequently disclosed the critical role of IRE1α/XBP1s arm of UPR in transducing adverse effect of particle in the CV system by regulating the transcription of ACE/ANGII/AT1R axis components in the vascular endothelium. These results not only provide previously unidentified evidence linking ER stress with particle toxicology, but also elucidate new mechanism for controlling vascular RAS activities by UPR-dependent transcriptional signals. In fact, we have observed that pretreatment of HUVECs with 4-PBA, the chemical inhibitor for ER stress, effectively inhibited IRE1α/XBP1s pathway activation, HIF1α accumulation, XBP1s/HIF1α complex formation and the augmentation of RAS components. Furthermore, PM2.5-induced proinflammatory mediators (chemokines and adhesion molecules) expression, adhesion of U937 cells to HUVECs and the production of oxidative stress mediators were also dramatically suppressed by 4-PBA co-treatment (Supplementary Fig. S4). Therefore, we conclude that governing IRE1α/XBP1s pathway activation may be effective to limit ANGII-dependent deleterious effects in CV system under particle exposure.

In summary, data in thisstudy have demonstrated that PM2.5 exposure induces endoplasmic reticulum (ER) instability, leading to the activation of IRE1α/XBP1s branch of UPR and links HIF1α transactivation to mediate ANGII-dependent endothelial dysfunction. Thus, identifying novel therapeutic targets to alleviate ER stress and restore local RAS homeostasis in the endothelium may be helpful for the management of CV burden under PM2.5 exposure.

## Methods

### Plasmids, antibodies and reagents

The HRE-driven luciferase reporter plasmid, in which the transcription of the luciferase reporter gene is driven by three repeated hypoxia-responsive elements, was provided by Dr. Chuanshu Huang (New York University). The siRNAs specific targeting PERK, ATF6, IRE1α, XBP1s and HIFα and their control siRNAs were designed and synthesized by Riobo Technology (Guangzhou, China). PMB, enalaipril and valsartan were purchased from Sigma-Aldrich (St. Louis, MO). The primary antibodies used in this study were purchased from Cell Signal Technology and Santa Cruz Biotechnology, including anti-AGT antibody (sc-7419), anti-ACE antibody (sc-20791), anti-AT1R antibody (sc-1173), anti-GRP78 antibody (CST, 3177), anti-ATF6 antibody (sc-166659), anti-IRE1α antibody (sc-3294), anti-XBP1s antibody (sc-7160), anti-phospho-PERK antibody (CST, 3179), anti-PERK antibody (CST, 5683), anti-HIF1α antibody (CST, 3716), anti-LaminA/C antibody (CST, 4777) and anti-ACTB antibody (sc-69879).

### Exposure of animals to PM2.5

Preparation of PM2.5 sample collected in Wuhan, China, and its water-soluble elemental components analysis were described previously^17^. Male Sprague-Dawley (SD) rats (7–8 weeks of age) were lightly anesthetized by an intraperitoneal injection of pentobarbital sodium (30 mg/kg weight, Sigma) and then intratracheally (IT) instilled with PM2.5 (1.5 mg/kg weight, in a 150 µL sterile saline suspension) every other day for 3 times. PM2.5 sample was sonicated for 5 min before IT instillation. Rats in the vehicle control group were IT with identical volume of sterile saline. After the last time IT instillation, all rats were allowed to recover for 24 hr before being executed.

The protocols of all animal experiments were approved by the Ethics Committee of Beijing Institute of Basic Medical Sciences. The experiments were performed in strict accordance with the National Institutes of Health Guidelines for the Care and Use of Laboratory Animals. Rats were sacrificed under anesthesia (10% chloral hydrate, peritoneal injection), and all efforts were made to minimize discomfort and pain.

### Endothelial cells culture and treatments

Human umbilical vein endothelial cells (HUVECs) were purchased from American Type Culture Collection (ATCC) and maintained in DMEM (Life Technologies) with 10% fetal bovine serum (Life Technologies) supplemented with antibiotic/antimycotic (Life Technologies). The primary rat aortic endothelial cells (RAECs) were isolated by collagenase digestion method as described previously^18^. Prior to cell exposure, particle samples were weighed, resuspended in cell culture medium, and diluted to the final concentrations of 6.25, 12.5 and 25 μg/mL for cell treatment after sonication for 5 min.

To test whether there is any contamination of endotoxin in PM2.5 samples to induce nonspecific up-regulation of RAS components in HUVECs, the cells were exposed to PM2.5 for 24 hr with or without co-treatment of PMB (50 μg/mL, Sigma-Aldrich), an antibiotic widely used to eliminate the effects of endotoxin contamination. To test the effect of ANGII/AT1R pathway activation on the induction of vascular endothelial cell dysfunction, HUVECs were pretreated with enalapril (100 μM) or valsartan (1 μM) for 2 hr and then subjected to PM2.5 exposure for 24 hr.

### siRNAs transfection

Cell transfections with siRNAs were performed with LipofectAMINE^TM^ RNAi MAX (Invitrogen) according to the manufacturer’s instructions. Regularly, the specific siRNA or its control siRNA was transfected into the cells with the final concentration of 50 nM and then the efficiency of gene silencing was detected 36 hr after transfection.

### Luciferase reporter assay

Cell transfections with the luciferase reporter plasmids were performed with LipofectAMINE 2000 (Invitrogen) according to the manufacturer’s instructions. Briefly, 2 × 10^5^ HUVECs were co-transfected with 2 μg of HRE-driven luciferase reporter and 0.1 μg of Renilla luciferase reporter as the internal control followed by subjecting to PM2.5 exposure. Luciferase activity was tested using Firefly-Renilla Dual-Luciferase Reporter Assay System (Promega). The data were obtained by normalizing the activity of the experimental reporter to that of the internal control. The results were presented as the relative induction by normalizing the luciferase activity in the PM2.5-treated cells to the luciferase activity in untreated control cells, as previously described^17^.

### RT-PCR assay

To analyze the induction of *AGT, ACE* and *AT1R* transcription in HUVECs and RAEC under PM2.5 exposure, total RNA was extracted with TRIzol reagent (Sigma-Aldrich) and cDNA was synthesized with the ThermoScriptTM RT-PCR system (Thermo Fisher Scientific). The oligonucleotides were synthesized and used as the specific primers to amplify human or rat *AGT, ACE* or *AT1R* cDNAs. The sequences of the primers used in RT-PCR assays were as follows: rat *AGT*: forward: 5′-gctggagctaaaggacacacag-3′, reverse: 5′-tggccagcacgagcttatct-3′; rat *ACE*: forward: 5′-gattgcagccgggcaactttt-3′, reverse: 5′-ccttcgcagttttggtcagt-3′; rat *AT1R*: forward: 5′-tcttctcaatctcgccttgg-3′, reverse: 5′-gacttcattgggtggacgat-3′; human *AGT*: forward: 5′-tcccctgtggatgaaaaggc-3′, reverse: 5′-ggaggtgcagttcttgtcct-3′; human *ACE*: forward: 5′- aacgccctgctaagcaacat-3′, reverse: 5′-tcgaaggtgggggagttgta-3′; human *AT1R*: forward: 5′-aagtcggcaccaggtgtattt-3′, reverse: 5′-aagcataagtcagccagtgct-3′. For quantitative evaluation of the mRNA levels of RAS components, the bands were scanned and the intensity was quantified by densitometric analysis using Gel-Pro Analyzer software. The amount of RAS components were normalized to an internal control (ACTB) and expressed as relative density of mRNA. Three independent experiments were performed, and the final quantitative results were presented as the mean ± SD.

### ChIP assay

ChIP assay was performed using Enzymatic Chromatin IP Kit (Cell Signal Biotechnology) according to the protocol recommended by the manufacturer. ChIP primers were designed to specifically amplify the regions covering the putative HIF1α- and XBP1-responsive elements within human *AGT, ACE* or *AT1R* promoter. The sequences of the primers used in ChIP assays were as follows: *AGT*: forward: 5′-attgttcagtcagtgaatgtacag-3′, reverse: 5′-actacacaggtcggaatttatgta-3′; *ACE*: forward: 5′-acttggagcaagcctctcaacctg-3′, reverse: 5′-cggtgtctcgcgtcctcgaaacct-3′; *AT1R*: forward: 5′-ttgctttctggcatcaacctcact-3′, reverse: 5′-actagagggttgagtacgatactc-3′.

### Western blot assay

Protein extracts of human or rat endothelial cells were prepared with ice-cold cell lysis buffer (20 mM Tris-HCl, 150 mM NaCl, 1 mM EDTA, 1 mM EGTA, 1% NP-40, 10% glycerol, 2.5 mM Na_4_P_2_O_7_, 0.5 mM Na_3_VO_4_, 1x proteinase inhibitors), resolved by SDS-PAGE and transferred to a PVDF membrane. After being blocked, the blots were probed with the appropriate primary antibodies and the horseradish peroxidase (HRP)-conjugated secondary antibodies (Cell Signaling Technology). Bands were detected as described in our previous report^17^.

### Immunoprecipitation

To detect the interaction between endogenous XBP1s and HIF1α in HUVECs under PM2.5 exposure, protein extracts of 1 × 10^7^ HUVECs were prepared with ice-cold cell lysis buffer (20 mM Tris-HCl, 150 mM NaCl, 1 mM EDTA, 1 mM EGTA, 1% NP-40, 10% glycerol, 2.5 mM Na_4_P_2_O_7_, 0.5 mM Na_3_VO_4_, 1x proteinase inhibitors) on ice. 500 μg total cell lysates were precleared by incubation with Protein A/G plus-agarose (Santa Cruz Biotechnology) and incubated with 2 μg anti-XBP1s antibody at 4 °C over night. Then 40 μl of Protein A/G plus-agarose was added into the mixture and incubated with agitation for an additional 4 hr at 4 °C. The immunoprecipitated samples were washed with the ice-cold cell lysis buffer for five times and then subjected to the western blot assay with the anti- HIF1α antibody.

### Cytoplasmic and nuclear proteins preparation

Cytoplasmic and nuclear proteins were prepared with the *ProteinExt* Mammalian Nuclear and Cytoplasmic Protein Extraction Kit (TransGen Biotech) according to the manufacturer’s protocols. Briefly, 1 × 10^7^ HUVECs were lysed with the 0.5 mL Cytoplasmic Protein Extraction Buffer (CPEB) on ice for 10 min and then the supernatants were collected (cytoplasmic proteins). After wishing with CPEB, the precipitants were incubated with 0.1 mL Nuclear Protein Extraction Buffer (NPEB) and shake vigorously on ice for 30 min to prepare the nuclear proteins.

### Immunofluorescence assay

To determine the surface level of AT1R, vascular cell adhesion molecule 1 (VCAM-1), endothelial-selectin (E-selectin) and platelet-selectin (P-selectin) on HUVECs and REAC, cells were incubated with FITC-conjugated antibodies (eBioscience). Stained cells were analyzed on a FACSCalibur using CELLQuest software (Becton Dickinson).

### ELISAs

Cytokines and peptide [ANGII, soluble intercellular adhesion molecule-1 (sICAM-1), interleukin 8 (IL-8) and monocyte chemoattractant protein-1 (MCP-1)] production in the cell culture supernatants of HUVECs and the rat serum ANGII levels were quantified with immunoassay kits (Jitai Biotechnology and Guduo Biotechnology) according to the manufacturers’ protocols.

### Monocytic adhesion assay

PM2.5-treated or untreated HUVECs were co-cultured with BCECF/AM (Life Technologies)-labeled monocytic U937 cells. The adhesion of U937 cells to the endothelial cells were evaluated either by photography under the confocal microscopy (ZEISS, LSM510 META) or flow cytometry (BD Biosciences, FACSCalibur)-based quantitative analysis.

### Oxidative stress assay

Reactive oxygen species (ROS) generation was determined by using the dye CM-H2DCFDA (Life Technologies), which can become fluorescent upon oxidation. Briefly, the working solution of the dye should be freshly prepared in DMSO before the experiments. To test the generation of ROS in PM2.5-challenged endothelial cells, cells were harvested and resuspended in the pre-warmed PBS buffer containing the final working concentration of 5 mM dye. The cells were incubated at room temperature for 30 min and then subjected to the low cytometry (BD Biosciences, FACSCalibur)-based quantitative analysis.

Malondialdehyde (MDA) was measured by the thiobarbituric acid (TBA) method with the materials provided in the Cell Malondialdehyde Assay Kit (Beyotime Technologies, China). Each sample was tested in triplicate. Three independent experiments were performed.

### Cell viability assay

Cell viability was determined by trypan blue exclusion assay after exposing HUVECs to PM2.5 for the different time periods. The amount of viable cells without trypan blue staining was counted. The quantitative results were averaged from three independent experiments.

### Statistics

All the data presented in this paper were collected from at least three independent experiments. The data were tested for significance by using Student’s t-test to determine the effect of treatment within a group or factorial design (AVONA) to determine the effects of treatment × group interactions. The results were presented as the mean ± SD. The level of significance was set at P < 0.05.

## Electronic supplementary material

Supplementary information
