# Supplementary material for: IRE1α/XBP1s branch of UPR links HIF1α activation to mediate ANGII-dependent endothelial dysfunction under particulate matter (PM) 2.5 exposure
Source: Sci Rep. 2017 Oct 18;7:13507. doi: 10.1038/s41598-017-13156-y (PMC5647447; doi:10.1038/s41598-017-13156-y)
Supplement: Supplementary file 1 — Supplementary information [file 41598_2017_13156_MOESM1_ESM.pdf]

IRE1α/XBP1s branch of UPR links HIF1α activation to mediate ANGII-dependent endothelial dysfunction under particulate matter (PM) 2.5 exposure

Xiuduan Xu[1, 2, #, $], Aodengqimuge[1,4, #], Hongli Wang[1, 5, #], Chen Xing[1], Ye Gu[1,6], Shasha Liu[1,6], Huan Xu[1, 2], Meiru Hu[1], Lun Song[*, 1, 2, 3]

[1]Department of Stress Medicine, Beijing Institute of Basic Medical Sciences, 27 Taiping Road, Beijing 100850; [2]Anhui Medical University, 81 Meishan Road, Hefei 230032; [3]Guangxi Medical University, 22 Shuangyong Road, Nanning 530021; [4]Department of New Drug Screening Center, China Pharmaceutical University, 24 Tongjiaxiang, Nanjing 210009; [5]Laboratory of Cellular and Molecular Immunology, School of Medicine, Henan University, 357 Ximen Road, Kaifeng 475004; [6]Department of Pathology, School of Basic Medical Sciences, Lanzhou University, Tianshui South Road, Lanzhou 730000; P. R. China.

Supplementary Materials and Methods

Antibodies

The primary antibodies used in the western blot assay were as follows: anti-ERK antibody (CST, 4695), anti-p38K antibody (CST, 9212), anti-ATF2 antibody (CST, 9226).

Cell treatment

To test whether inhibiting UPRs activation shows any effect on ANGII/AT1R pathway activation and vascular endothelial cell dysfunction in HUVECs induced by PM2.5, the cells were pretreated with 4-PBA (10 mM), the chemical inhibitor for ER stress, for 2 hr and then subjected to PM2.5 exposure for 24 hr.

Determination of whole cell proteins

After treating with PM2.5 for the different time periods, HUVECs were harvest and the variable cells were determined by trypan blue exclusion assay. Then whole cell extracts were prepared with ice-cold cell lysis buffer and then the concentration of cellular protein was determined by BCA Protein Assay Kit (Thermo Fisher Scientific). The results were presented as ug total proteins/10^5 cells.

XBP1 splicing assay

To analyze the induction of *XBP1* splicing in HUVEC under PM2.5 exposure, total RNA was extracted with TRIzol reagent (Sigma-Aldrich) and cDNA was synthesized with the ThermoScriptTM RT-PCR system (Thermo Fisher Scientific). The following oligonucleotides were synthesized and used as the specific primers to

amplify human *XBP1u* and *XBP1*s cDNAs: forward: 5’- CCTTGTAGTTGAGAACC AGGAG -3’, reverse: 5’- GGTCCAAGTTGTCCAGAATGC -3’.

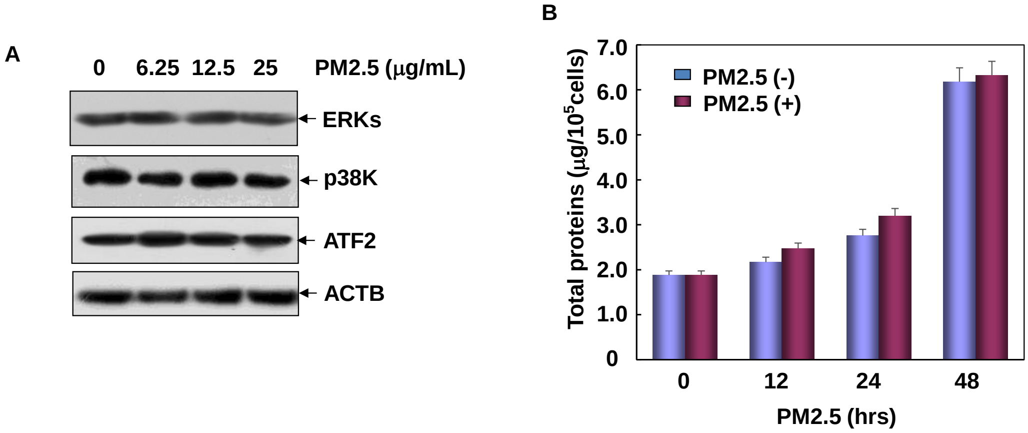

Supplementary Figure S1: PM2.5 exposure did not result to the increase of bulk rate of protein syntheses in HUVECs. (A) HUVECs were treated with different doses of PM2.5 for 24 hr and then the expression of p38K, ATF2 and ERK were determined. (B) HUVECs were treated with PM2.5 (12.5 μg/mL) for the indicated time periods and then the whole cell extracts were prepared to determine the total proteins.

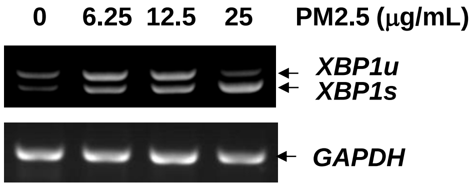

Supplementary Figure S2: PM2.5 exposure enhanced *XBP1* transcription and *XBP1u* mRNA spilcing in HUVECs. HUVECs were treated with different doses of PM2.5 for 24 hr and then the mRNA levels of *XBP1u* and *XBP1s* were detected.

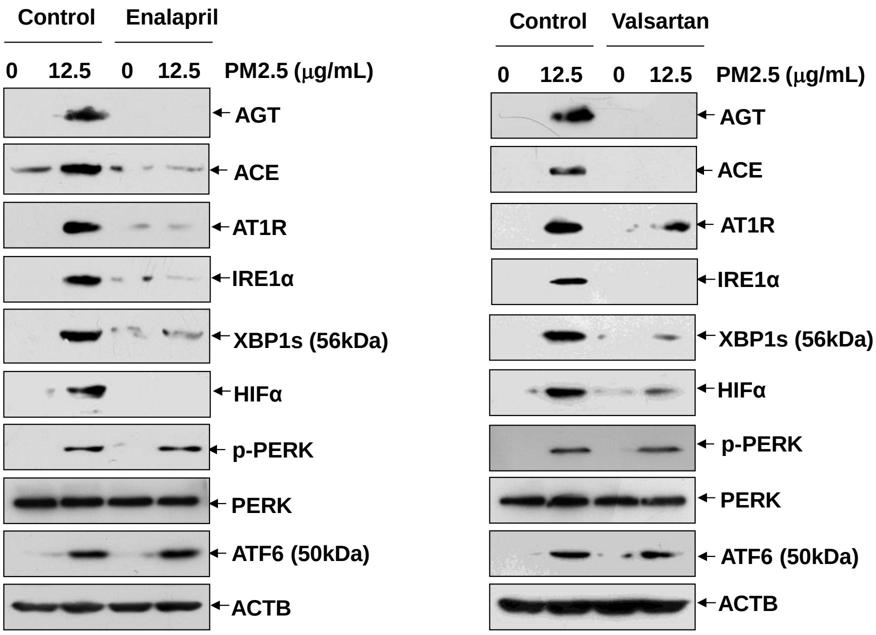

Supplementary Figure S3: Inhibiting ANGII production or blocking AT1R activation effectively suppressed IRE1/XBP1s/HIF1α cascade activation in HUVECs under PM2.5 exposure. HUVECs were pretreated with enalapril (100 μM) (A) or valsartan (1 μM) (B) for 2 hr and then subjected to PM2.5 exposure for 24 hr. The activation of three branches of UPR, the accumulation of HIF1α and the induction of RAS components expression were determined.

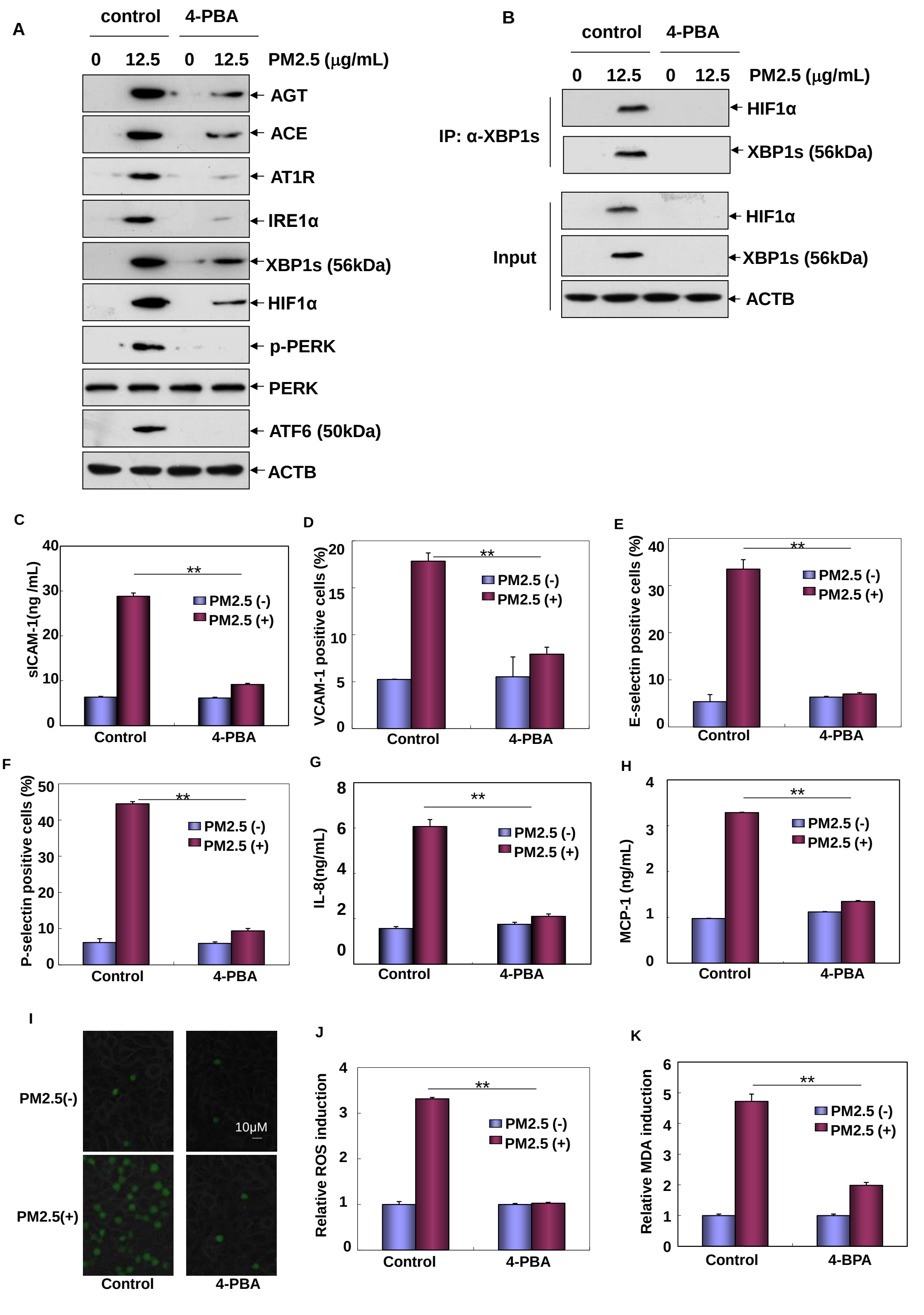

Supplementary Figure S4: Alleviating ER stress inhibited RAS-related endothelial cell dysfunction induced by PM2.5. (A) HUVECs were pretreated with 4-PBA (10 mM) for 2 hr and then subjected to PM2.5 exposure for 24 hr. The activation of three branches of UPR, the accumulation of HIF1α and the induction of RAS components expression were determined. (B) HUVECs were treated as described in A and then the interaction of XBP1s and HIF1α was determined by immunoprecipitation. (C-K) HUVECs were treated as described in A and then the expression of the proinflammatory mediators, adhesion of U937 cells to HUVECs and generation of oxidative stress mediators were determined (**, *P*<0.01).
